# The Role of *n*-3 Polyunsaturated Fatty Acids in the Prevention and Treatment of Breast Cancer

**DOI:** 10.3390/nu6115184

**Published:** 2014-11-18

**Authors:** Jiajie Liu, David W. L. Ma

**Affiliations:** Department of Human Health & Nutritional Sciences, College of Biological Sciences, University of Guelph, Guelph, ON N1G 2W1, Canada; E-Mail: jliu22@uoguelph.ca

**Keywords:** *n*-3 polyunsaturated fatty acids (PUFA), eicosapentaenoic acid (EPA), docosahexaenoic acid (DHA), α-linolenic acid (ALA), mammary gland, breast cancer (BC)

## Abstract

Breast cancer (BC) is the most common cancer among women worldwide. Dietary fatty acids, especially *n*-3 polyunsaturated fatty acids (PUFA), are believed to play a role in reducing BC risk. Evidence has shown that fish consumption or intake of long-chain *n*-3 PUFA, such as eicosapentaenoic acid (EPA) and docosahexaenoic acid (DHA), are beneficial for inhibiting mammary carcinogenesis. The evidence regarding α-linolenic acid (ALA), however, remains equivocal. It is essential to clarify the relation between ALA and cancer since ALA is the principal source of *n*-3 PUFA in the Western diet and the conversion of ALA to EPA and DHA is not efficient in humans. In addition, the specific anticancer roles of individual *n*-3 PUFA, alone, have not yet been identified. Therefore, the present review evaluates ALA, EPA and DHA consumed individually as well as in *n*-3 PUFA mixtures. Also, their role in the prevention of BC and potential anticancer mechanisms of action are examined. Overall, this review suggests that each *n*-3 PUFA has promising anticancer effects and warrants further research.

## 1. Introduction

Breast cancer (BC) is a major health problem among women worldwide, and is the second leading cause of death for women in Canada and the United States [1,2]. On average, 65 Canadian women will be diagnosed with BC per day, with 1 in 9 females expected to develop BC in their lifetime [1,2]. Both genetic and environmental factors are believed to play a role in a woman’s risk of developing BC [3,4]. Most anticancer drugs, developed to date, aim to kill cancer cells and decrease tumor burden but are relatively ineffective against some phases of tumorigenesis [5,6]. Thus, alternate strategies to prevent tumorigenesis are urgently required. In the past few decades, epidemiological studies have suggested that a healthy diet and lifestyle are critical for the prevention of BC. Dietary fatty acids are one of the most intensively studied dietary factors [7,8,9].

Saturated fatty acids (SFA), monounsaturated fatty acids (MUFA) and trans fatty acids (TFA) have been found to increase cancer risk; while specific polyunsaturated fatty acids (PUFA) are indicated to have anticancer effects [9,10]. There are two major classes of PUFA: *n*-6 PUFA and *n*-3 PUFA. In mammals, *n*-6 and *n*-3 PUFA are both essential fatty acids for health and must be consumed as part of the diet because they cannot be endogenously synthesized [11]. Linoleic acid (LA, 18:2*n*-6) and arachidonic acid (AA, 20:4*n*-6) are the two most common *n*-6 PUFA in typical Western diets; LA can be found in some plant oils such as corn and safflower oils, and AA usually comes from dietary animal sources or can be synthesized from LA [12,13]. α-linolenic acid (ALA, 18:3*n*-3) is the precursor of the *n*-3 PUFA family which can be further elongated and desaturated to two important long chain *n*-3 PUFA, eicosapentaenoic acid (EPA, 20:5*n*-3) and docosahexaenoic acid (DHA, 22:6*n*-3) [11]. ALA is a plant-derived *n*-3 PUFA, which is present in flaxseed, canola and soybean oils [14]. The longer chain *n*-3 PUFA, EPA and DHA can be obtained directly from marine sources such as seafood and fish oils, and are widely known for their cardioprotective benefit [13]. ALA is the major *n*-3 PUFA consumed in the Western diet, whereas intakes of EPA and DHA are typically low. It is estimated that the typical North American diet provides approximately 1.4 g of ALA and 0.1–0.2 g of EPA plus DHA per day [15]. With regard to dietary reference intake of *n*-3 PUFA, the Institute of Medicine (IOM) recommends since 2005 a daily intake 1.1 g ALA for women and 1.6 g ALA for men to prevent some chronic diseases, and up to ten percent of this can be consumed as EPA and/or DHA [16]. While current intakes meet IOM recommendations, in a 2014 report by the Academy of Nutrition and Dietetics, 500 mg EPA plus DHA per day is required for the general healthy adult population [17].

Results from both *in vivo* and *in vitro* studies suggest that *n*-6 PUFA accelerate tumorigenesis, in contrast, *n*-3 PUFA may have anticancer effects [18,19,20,21,22]. Western diets are typically deficient in *n*-3 PUFA and high in *n*-6 PUFA compared with traditional Asian diets [8,23,24]. Migration studies have shown that Asian women, who typically have a lower rate of BC and higher fish consumption exhibited an increased incidence of BC within one generation after migration to the Western countries [24,25]. Historically, the intake of *n*-6 and *n*-3 PUFA has been estimated to be approximately equal. However, in recent years, the content of Western diets has significantly increased in *n*-6 PUFA resulting in an increase in the *n*-6/*n*-3 PUFA ratio [8]. Excessive amounts of *n*-6 PUFA and a very high *n*-6/*n*-3 ratio (16:1 or higher), as is currently found in Western diets, have been suggested to promote the pathogenesis of many diseases such as cardiovascular disease, autoimmune diseases and some types of cancer; whereas increased levels of *n*-3 PUFA (a low *n*-6/*n*-3 ratio) have been shown to exert suppressive effects [8]. However, it remains to be resolved whether it is simply the reduced *n*-3 PUFA or the changing *n*-6/*n*-3 ratio that is relevant to these outcomes.

Substantial evidence from cell culture and rodent studies indicate that increased fish consumption or intake of *n*-3 PUFA inhibits BC cell proliferation and reduces BC risk relative to *n*-6 PUFA [26,27,28,29,30]. Nevertheless, there has been longstanding controversy in epidemiological and observational studies regarding the potential anticancer effects of *n*-3 PUFA due to the inability to show causality. Previous published reviews have focused on the role of fish and marine *n*-3 fatty acids in BC prevention, whereas the evidence for ALA and individual effects of long chain *n*-3 PUFA is lacking [3,8,11,23,31,32,33,34]. Since a typical North American diet is mainly comprised of ALA as the source of *n*-3 PUFA, it is necessary to elucidate the specific effects of ALA and cancer risk. Therefore, the purpose of the present review is to evaluate the preventative role of ALA, EPA and DHA in BC development when consumed individually, as well as in *n*-3 PUFA mixtures through dietary and supplemental forms. In addition, the potential mechanisms by which they exert anticancer effects will also be discussed.

## 2. The Effects of *n*-3 PUFA in Human BC Studies

Dietary *n*-3 PUFA may influence breast cancer (BC) progression and prognosis. In the past ten years, six prospective cohort studies (Table 1) and nine case-control studies (Table 2) have examined the association between the consumption of either fish or fish oil supplements and BC risk, showing a protective effect of *n*-3 PUFA. These studies were conducted in many different geographic areas with mixed findings. In general, Asian populations with a low total fat intake and high fish consumption, associated *n*-3 PUFA intake with a reduced risk of BC [35,36,37,38,39]. There was also a weak association with reduced BC risk in US studies involving women whose diets had higher *n*-6 PUFA content combined with fish oil supplements [18,40,41]. European studies were less consistent and somewhat contradictory [42,43,44,45].

In the Japan Collaborative Cohort (JACC) study, a significant decrease in the risk of BC was detected in women with the highest dietary intake of fish fat and the long-chain *n*-3 PUFA [35]. Similar results were observed in a large prospective study of 35,298 Singapore women, indicating an inverse association between dietary *n*-3 PUFA from marine sources and BC risk [36]. Relative to the lowest quartile of *n*-3 PUFA intake, individuals in the top three quartiles exhibited a 26% reduction in BC risk (relative risk = 0.74; 95% confidence interval = 0.58–0.94). Additionally, a recent analysis from the VITamins And Lifestyle (VITAL) cohort carried out in US indicated that the current use of fish oil supplements was associated with a decreased risk of localized invasive ductal carcinomas in postmenopausal women [40].

**Table 1:** *n-*3 PUFA and breast cancer risk: Prospective cohort studies.

Year	Country	Subjects	Method of Assessment	*n-*3/*n*-6 PUFA Source	BC Risk	Reference
2005	Japan	26,291 women 40–79 years 129 BC cases	FFQ ^1^	Animal and fish fat, vegetable oil, SFA, MUFA and PUFA	↑ fish fat, EPA + DHA ↓ BC risk	[35]
2003	Singapore	35,298 women 45–74 years 342 BC cases	FFQ	Fish/shellfish, saturated, monounsaturated and polyunsaturated fat	↑ *n*-3 PUFA from fish/shellfish ↓ BC risk ↑ *n*-6 PUFA ( low marine *n*-3) ↑ BC risk	[36]
2010	US	35,016 postmenopausal 50–76 years 880 BC cases	FFQ	Dietary fish oil supplement	↑ fish oil ↓ risk of invasive ductal carcinomas	[40]
2009	France	56,007 women 40–65 years 1650 BC case	FFQ	ALA and *n*-6 PUFA from fruit, nuts and vegetable oils; Long chain *n*-3 PUFA from meals	no association between total *n*-3 and BC risk ↑ ALA ↓BC risk ↑ long chain *n*-3 PUFA ↓ BC risk (at highest quintile of *n*-6 PUFA)	[42]
2003	Denmark	23,693 postmenopausal 50–64 years 424 BC cases	FFQ	Fish	↑ intake of fish ↑ ER + BC incidence	[43]
2011	China	72,571 women 40–70 years 712 BC cases	FFQ	Fish, marine-derived *n*-3 PUFA red meat	↑ *n*-6/*n*-3 PUFA ratio ↑ BC risk	[37]

^1^ FFQ: food frequency questionnaire; ↑: increase; ↓: decrease.

**Table 2:** *n*-3 PUFA and breast cancer risk: Case-control studies.

Year	Country	Subjects Characteristics	Method of Assessment	*n*-3/*n*-6 PUFA Source	BC Risk	Reference
2007	Japan	103 incident BC cases 309 controls	erythrocyte membrane FFQ	dietary food intake including soy and meat products, fish and other seafood, vegetables	↑ dietary intake of *n*-3 fatty acids ↓ BC risk ↑ long chain *n*-3 PUFA in erythrocyte ↓ BC risk ↑ saturated fatty ↑ BC risk	[46]
2007	China	322 incident BC cases 1030 controls	erythrocyte membrane		↑ total *n*-3 fatty acids and EPA ↓ BC risk	[47]
2009	China	155 NPFC ^1^ 185 PFC ^2^ 241 BC, 1030 controls	erythrocyte membrane FFQ	dietary food intake	↑ EPA ↓ risk of NPFC ↓ progression of PFC to BC ↑ γ-linolenic acid ↑ risk of NPFC, PFC and BC	[38]
2002	US	73 BC patients 74 controls	breast adipose tissue		↑ EPA and DHA ↓ *n*-6/*n*-3 PUFA ratio ↓ BC risk ↑ *n*-6 PUFA ↑ BC risk	[18]
2003	US	565 incident BC 554 controls	FFQ	daily fat intake	↓ *n*-6/*n*-3 PUFA ratio ↓ BC risk (premenopausal) ↑EPA, DHA ↓ BC risk (21% and 18%, respectively)	[41]
2009	Denmark	463 BC cases 1098 controls	Gluteal adipose tissue biopsy	dietary food intake	No association between total or individual marine *n*-3 PUFA in adipose tissue and risk of BC	[44]
2002	France	241 invasive BC cases 88 controls-benign breast disease	breast adipose tissue		↑ ALA ↑ DHA ↓ *n*-6/*n*-3 PUFA ratio ↓ BC risk	[45]
2012	Mexican	1000 incident BC cases 1074 controls	Interview and FFQ	dietary food intake	↑ *n*-3 PUFA ↓ BC risk (obese women) ↑ *n*-6 PUFA ↑ BC risk (premenopausal)	[19]
2009	South Korea	358 incident BC patients 360 controls	FFQ	fatty and lean fish	↑ fatty fish consumption ↓ BC risk ↑EPA and DHA derived from fish ↓ BC risk	[39]

^1^ Benign proliferative fibrocystic conditions (PFC); ^2^ non-proliferative fibrocystic conditions (NPFC); ↑: increase; ↓: decrease.

The association between dietary intakes of *n*-3 PUFA or fish oil supplements with overall survival was also examined. Patterson *et al.* demonstrated that women with higher intakes of EPA and DHA from food, but not from fish oil supplements, had a dose-dependent reduction in all-cause mortality [48]. They also showed a reduced risk of additional BC events of approximately 25% when compared with the lowest tertile of intake (tertile 3:hazard ratio = 0.72; 95% confidence interval = 0.57–0.90) [48]. In support of these self-reported intake studies, Zheng *et al.* performed a comprehensive analysis of 21 independent prospective cohort studies and found that marine *n*-3 PUFA were associated with a 14% risk reduction of BC, and the relative risk remained similar whether marine *n*-3 PUFA was measured as dietary intake or as tissue biomarkers [7]. Further, a dose-response analysis indicated a 5% lower risk of BC per 0.1 g/day (0.95, 0.90 to 1.00, *I*^2^ = 52%) increment of dietary marine *n*-3 PUFA [7]. Conversely, a French study comprising over 56,000 women found no association between total *n*-3 or *n*-6 PUFA intake and BC risk [42]. Also, a large study of postmenopausal women in Denmark concluded that increased fish consumption was associated with elevated incidence rates of BC, but this association was present only for development of estrogen positive BC [43]. These null studies were mostly conducted in European populations with relatively low per capita intake of *n*-3 PUFA [32,44].

In order to examine the relationship between *n*-3 PUFA exposure and BC risk, several case-control studies have been conducted using different biomarkers (Table 2). Kuriki *et al.* investigated the fatty acid compositions of erythrocyte membranes as a biomarker and demonstrated that BC risk exhibited a significant inverse association with dietary intake of *n*-3 PUFA derived from fish and high levels of long-chain *n*-3 PUFA in erythrocyte membranes [46]. Another assessment of erythrocyte fatty acid composition found the inverse association significant only for EPA and total *n*-3 PUFA content [47]. Furthermore, Shannon *et al.* evaluated the role of *n*-3 and *n*-6 PUFA in the development of benign proliferative fibrocystic conditions (PFC) and non-proliferative fibrocystic conditions (NPFC) in the breast [38]. They showed that women in the highest quartile of erythrocyte EPA concentrations were 67% less likely to have NPFC alone or with BC, and EPA significantly lowered the risk of progressing from PFC to BC by 43% [38]. However, γ-linolenic acid (*n*-6 PUFA) was found to be positively associated with nearly all conditions [38]. These results were consistent with an earlier meta-analysis showing that total and individual *n*-3 PUFA, especially EPA and DHA, play a protective effect against BC, while total SFA, MUFA, palmitic and oleic acids were associated with increased BC risk [9].

In a Korean case control study, 358 patients with BC and 360 healthy controls underwent dietary assessment by questionnaire and interview to determine their dietary consumption of fish and *n*-3 PUFA derived from fish. Both pre- and postmenopausal women in the highest quartile of fatty-fish intake had a lower incidence of BC (odds ratio OR = 0.23, 95% CI = 0.13–0.42; *p* < 0.001), but the protective effect of EPA and/or DHA intake was only observed for postmenopausal women [39]. These findings were similarly observed in a study of Mexican women where BC risk was lower in obese women (BMI ≥ 30) with high *n*-3 PUFA intake, not in women of normal weight [19]. In contrast, a case-cohort study of Danish women did not find any association between either total or individual marine *n*-3 PUFA intake and BC risk [44]. As the total levels of marine *n*-3 PUFA intake were low in Europe, this may account for observed discrepancies relative to populations consuming marine-rich diets [32,44].

Other population studies have investigated interactions between *n*-3 and *n*-6 PUFA. Quantifying consumption of both types of fatty acids is an essential step in isolating *n*-3 PUFA specific effects. The study of Bagga *et al.* showed that excessive intake of *n*-6 PUFA contributed to the high risk of BC in US, while a decreased risk of BC development was accompanied with higher EPA and DHA consumption [18]. A similar inverse relationship was also observed in regard to the *n*-6/*n*-3 PUFA ratio. In another US study, when the analysis was restricted to pre-menopausal women, the consumption of the lowest ratio of *n*-6 to *n*-3 was associated with a 41% reduction of BC risk, although it was not significant [41]. This observation was also observed in studies conducted in China and France, although there was no association between *n*-3 PUFA intake and BC risk, low *n*-3 PUFA intake by women who had the highest *n*-6 PUFA was correlated with elevated BC risk [37,45]. However, based on the ratio, it is not possible to determine whether it is increased *n*-6 or decreased *n*-3 PUFA that is the causal driver of BC risk. Nevertheless, these studies indicate the necessity of higher *n*-3 PUFA intakes given that *n*-6 intakes are adequate in all populations, and thus heightening the potential value of *n*-3 PUFA as effective agents against BC.

Diet intervention by *n*-3 PUFA supplements as a mean of decreasing BC risk in women still needs to be tested clinically. To date, few human intervention studies have assessed the effectiveness of *n*-3 PUFA in BC prevention and treatment. One randomized clinical trial tested the combined effects of *n*-3 PUFA (EPA + DHA = 3.36 g/day, 2 year) and Raloxifene (anti-estrogen) in reducing risk of BC in postmenopausal women [49]. Although the plasma *n*-6/*n*-3 ratio significantly decreased among subjects after *n*-3 PUFA intervention compared with the subjects without intervention, *n*-3 PUFA administration did not affect any selected biomarkers that associated with BC risk [49]. While in another human intervention study, Thompson *et al.* demonstrated that daily intake of 25 g flaxseed (ALA = 57% of total fatty acids) can significantly reduce cell proliferation and increase cell apoptosis in tumors of postmenopausal BC patients. These limited results provide encouragement for future study of *n*-3 PUFA as an adjuvant therapy in BC [50].

## 3. PUFA—Potential Mechanisms of Action

For more than 30 years, numerous studies have attempted to establish whether there is a causal relationship between *n*-3 PUFA ingestion and a reduction in mammary carcinogenesis. Mounting evidence shows that dietary *n*-3 PUFA may exert an anti-carcinogenic action by altering the composition of cell membrane phospholipids, inhibiting AA metabolism and decreasing AA derived eicosanoids, as well as modulating the expression and function of numerous receptors, transcription factors and lipid derived signaling molecules. However, studies of the effects of dietary *n*-3 PUFA on BC progression and prognosis are limited in humans. Epidemiological studies do not allow for the analysis of important cellular interactions and the specific molecular pathways which are activated during the course of tumor initiation and cancer progression in the mammary gland. Thus, the use of animal models (*in vivo*) and BC cell lines (*in vitro*) provide crucial avenues for improving our understanding of the underlying biological pathways involved in trigger BC development and potential therapeutic approaches for BC treatment and prevention. Here we first introduce some potential mechanisms and important downstream mediators that involved in the anticancer action of *n*-3 PUFA.

### 3.1. Influence on Cell Plasma Membrane Composition

Fatty acids play an important role in membrane biogenesis in the form of glycerophospholipids, a major class of lipids found all cell membranes [51]. Dietary PUFA integrate into plasma membrane glycerophosholipids and influence the fatty acid composition [52]. The *sn*-1 position on the glycerol backbone of glycerophospholipids is usually linked to saturated fatty acids, and the *sn*-2 position is linked to an *n*-6 PUFA such as AA. Increased intake of dietary *n*-3 PUFA may replace *n*-6 with *n*-3 fatty acids at the *sn*-2 position of glycerophospholipids [52]. Since *n*-3 PUFA has a greater density compared to *n*-6 PUFA, the aggregation of *n*-3 fatty acids tend to be closer to the lipid-water interface of the membrane. This characteristic can significantly affect plasma membrane fluidity and permeability [53]. In addition, due to the high level unsaturation of long chain *n*-3 PUFA, they have very poor affinity for cholesterol [52,54]. Membrane cholesterol serves as a spacer for the hydrocarbon chains of sphingolipids and maintains the assembled microdomains of lipid rafts [55]. Thus, cholesterol depletion leads to the disorganization of lipid raft structure [54]. Lipid rafts are important membrane domains for cell signaling since it is enriched with many regulatory proteins and some growth factor receptors. [54,55] As a result, the incorporation of *n*-3 PUFA, especially EPA and DHA, can disturb formation of lipid rafts and suppress raft-associated cell signal transduction [22,56,57].

### 3.2. Inhibition of Arachidonic Acid (AA) Derived Eicosanoid Biosynthesis

**Figure 1:**
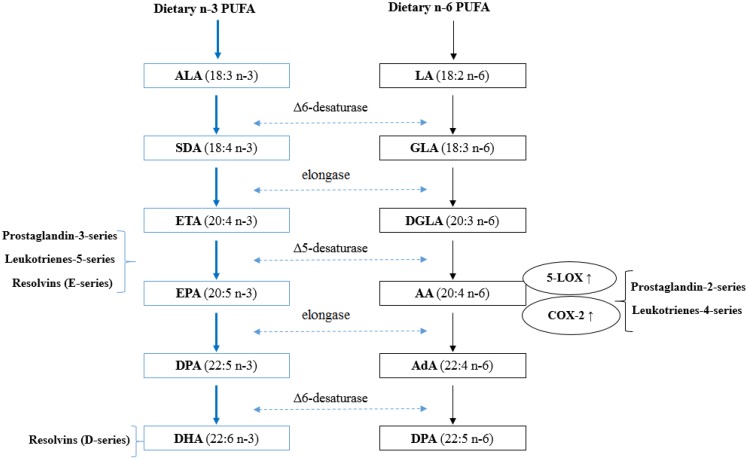
Synthetic pathways of long-chain PUFA and eicosanoids. α-linolenic acid (ALA; 18:3*n*-3) and linoleic acid (LA; 18:2*n*-6) are essential PUFA obtained from the diet, and involve in similar sequential desaturation and elongation steps, give rise to long chain, more unsaturated PUFA eicosapentaenoic acid (EPA; 20:5*n*-3), docosahexaenoic acid (DHA; 22:6*n*-3), and arachidonic acid (AA; 20:4*n*-6). Relevant intermediates in these pathways include SDA (stearidonic acid), ETA (eicosatetraenoic acid), DPA (docosapentaenoic acid), GLA (γ-linolenic acid), DGLA (dihomo-γ-linolenic acid) and AdA (adrenic acid). Both AA and EPA are substrates for the synthesis of eicosanoid products such as prostaglandins (PG) and leukotrienes (LT). The products of *n*-6 PUFA tend to promote cell proliferation while the products of *n*-3 PUFA have anti-tumorigenic properties. *n-*3 PUFA may lower the risk of BC by disrupting the biosynthesis of AA-derived inflammatory eicosanoids.

One of the key cellular functions of PUFA is related to their enzymatic conversion into eicosanoids. Eicosanoids are short-lived, hormone-like lipids, typically comprised of 20 carbon atoms, which play a critical role in platelet aggregation, cellular growth and cell differentiation [31]. The most salient mechanism by which *n*-3 PUFA reduce tumor development is through inhibiting the synthesis of inflammatory eicosanoids derived from AA [31]. As indicated in Figure 1, firstly, both ALA and LA are initially converted to their long-chain metabolites (EPA and AA, respectively) through the same desaturation/elongation pathway; therefore, there exists potential competition between these two families of fatty acids for desaturases and elongases. The initial conversion of ALA to stearidonic acid (18:4*n*-3) is the rate limiting reaction of the pathway. The affinity of delta 6-desaturase for ALA is greater than for LA [58]. As a result, a higher intake of ALA reduces the synthesis of AA from LA and thus, less AA is available for synthesis of inflammatory eicosanoids [59]. Secondly, increase consumption of *n*-3 PUFA results in their incorporation into membrane phospholipids, where they partially replace AA, therefore reducing the substrate for AA derived eicosanoids. Healy *et al.* showed that dietary supplements with four different concentrations of fish oil resulted in the incorporation of EPA and DHA into human inflammatory cells occurs in a dose-response fashion at the expense of AA [60]. Thirdly, both AA and EPA are substrate for eicosanoid synthesis such as prostaglandins (PG) and leukotrienes (LT) [31]. AA can be metabolized by two major pathways including the cyclooxyenase (COX) and lipoxygenase (LOX) pathways. COX-2 catalyzes the rate limiting step in the formation of 2-series PGs. 5-LOX catalyzes the first step in oxygenation of AA to produce hydroxyl derivatives and 4-series LTs. The overexpression of COX-2 has been detected in many types of cancers and PGE_2_ has been shown to promote cell proliferation in mammary tumor tissues [61]. PGE_2_ stimulates the expression and activation of aromatase, the enzyme that converts androgens to estrogens [62]. Therefore, it is hypothesized that estrogen levels can be lowered by decreasing *n*-6 PUFA intake. In contrast, EPA metabolism results in 3-series PGs and 5-series LTs, with a slightly different structure and anti-tumorigenic properties [58]. *n*-3 PUFA supplementation has been shown to lower production of PGE_2_ (by 60%) and LTB_4_ (by 75%) in human peripheral blood mononuclear cells [63]. Furthermore, *n*-3 PUFA has been suggested to suppress the expression of COX-2 and 5-LOX. Feeding mice with high *n*-3 PUFA diets influences mammary tumor development by down-regulating COX-2 and 5-LOX expression [20,64]. In addition to the inhibitory effects on the generation of inflammatory eicosanoids, recent studies have identified a novel group of lipid mediators, termed resolvins, which are formed from EPA and DHA [65,66]. These mediators appear to exert potent anti-inflammatory actions and are considered as potential therapeutic interventions for some chronic inflammatory diseases and cancer [67,68].

In general, *n*-3 PUFA can not only block AA metabolism, but can also compete with AA for eicosanoid synthesis. This anti-inflammatory effect of *n*-3 PUFA is of interest since chronic inflammation has been linked to cancer initiation and progression [69].

### 3.3. Influence on Gene Expression, Transcription Factor Activity and Signal Transduction

**Figure 2:**
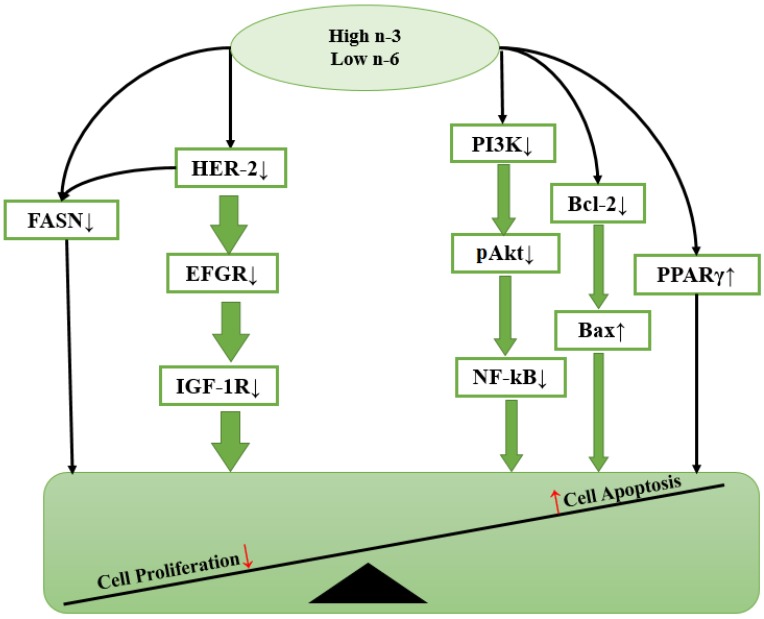
Hypothetical scheme showing how *n*-3 PUFA modulates cell functions via intracellular signaling molecules. Cell proliferation and cell apoptosis are the two important fundamental processes integral to carcinogenesis. *n-*3 PUFA exerts anti-cancer effects by reducing the expression of some growth factors including human epidermal growth factor receptor-2 (HER-2), epidermal growth factor receptor (EGFR) and insulin-like growth factor 1(IGF-1R); inhibiting cell proliferation by either activating PPARγ or decreasing levels of fatty acid synthase (FAS) protein; and promoting cell apoptosis via blocking PI3K/Akt pathways, downregulating phosphorylated Akt, inhibiting NF-κB activity and lowering Bcl-2/Bax ratio.

BC development is a multi-step process that requires the accumulation of several genetic alterations in a single cell. Many studies have now demonstrated that the regulation of intracellular signaling is a critical aspect of controlling cell functions in various types of cancers. Neoplastic growth and progression are generally regarded as dependent on a high rate of cell proliferation and a low rate of apoptosis. Dietary PUFA and their metabolites may exert some of their anti-cancer or tumor-promoting effects by affecting gene expression or activating signal transduction molecules involved in the control of cell proliferation, differentiation apoptosis and metastasis (Figure 2).

#### 3.3.1. EGFR and HER-2

Among numerous factors, carcinogenesis involves the activation of oncogenes such as the epidermal growth factor receptor (EGFR) and human epidermal growth factor receptor-2 (HER-2). EGFR is a receptor tyrosine kinase, which plays essential roles in regulating a number of cellular processes including cell proliferation, survival and migration [70]. EGFR is usually activated in response to extracellular ligands (epidermal growth factor [EGF)) by its phosphorylation [71]. Dysregulated EGFR activation is often associated with overexpression of EGFR, which has been observed in several cancer types including breast carcinomas [72]. Marine *n*-3 PUFA were able to inhibit EGFR activity, in particular, DHA was found to induce apoptosis in BC cells by down-regulating EGFR expression [71]. Similar to EGFR, HER-2 (HER-2/neu or erbB-2) is a 185-kD transmembrane receptor tyrosine kinase that is involved in human mammary oncogenesis [73]. The overexpression of HER-2 occurs in 25%–30% of human invasive BCs, and is associated with a more aggressive phenotype and poor patient prognosis [12,74]. HER-2 functions as a co-receptor and forms homodimers or heterodimers with EGFR or insulin-like growth factor 1(IGF-1R) to activate downstream target signaling cascades that involve cell survival and proliferation, such as phosphatidylinositol 3-kinase (PI3K)/Akt, mitogen-activated protein kinase (MAPK) and inhibition of apoptotic pathways such as Bcl-2-associated death promoter protein [75,76]. It should be noted that the HER-2 receptors also activate lipogenic pathways mediated by the fatty acid synthase (FAS) protein [6], a key lipogenic enzyme catalyzing the terminal steps in the de novo biogenesis of fatty acids in cancer pathogenesis [51]. Dietary *n*-3 PUFA were demonstrated to inhibit the early stages of HER-2/neu-mediated mammary carcinogenesis in rats [77]. Notably, ALA alone was able to reduce HER-2 protein expression by 79% in MCF-7 cell lines [78]. Both EGFR and HER-2 are regarded as important therapeutic targets against BC, and *n*-3 PUFA may be a dietary treatment for controlling the growth factor-mediated oncogenesis.

#### 3.3.2. Peroxisome Proliferator-Activated Receptor Gamma (PPARγ)

Peroxisome proliferator-activated receptors (PPARs) are members of the nuclear receptor superfamily and function as ligand-activated transcription factors [79]. PPARγ is a subset of the PPAR family, it is mainly expressed in adipose tissue, mammary gland, colon and the immune system [80,81]. PPARγ regulates the expression of target genes by binding to DNA sequence elements, termed PPAR response elements (PPREs). PPREs have been identified in the regulatory regions of a variety of genes that are involved in lipid metabolism and homeostasis, but recently have appeared to be involved in cell proliferation, cell differentiation, and inflammatory responses [79,82]. PPARγ ligands include naturally occurring compounds such as PUFA and eicosanoids, as well as synthetic activators, such as the hypolipidemic drugs [83]. Clay *et al.* indicated that induction of apoptosis is a biological response resulting from PPARγ activation in some BC cells [84]. *n-*3 PUFA are direct agonists for PPARγ, which have been shown to exert anti-tumorigenic effects via the activation of PPARγ [82,85]. For instance, DHA was found to attenuate MCF-7 cell proliferation by activation of PPARγ [86]. In addition, dietary supplementation with a low ratio of *n*-6/*n*-3 PUFA (1:14.6) was shown to increase PPARγ protein content, which was paralleled with a reduction of tumor burden in rats with induced mammary carcinogenesis [87]. As a result, PPARγ activation is beneficial for controlling BC, which suggests a potential role for PPARγ ligands in the treatment of BC.

#### 3.3.3. Bax/Bcl-2

Apoptosis is a form of cell death triggered during a variety of physiological conditions and is tightly regulated by a number of gene products that promote or block cell death at different stages [88]. Bcl-2 is well-known as an important apoptosis-regulator protein [89], normally blocking apoptosis and its overexpression contributes to BC by prolonging cell survival [90]. Bax is a pro-apoptotic member of the Bcl-2 family of proteins. It is likely to have pore-forming activity to increase mitochondrial membrane permeability, and can also form a homodimer with Bcl-2 to enhance the effects of apoptotic stimuli [90,91]. Raisova *et al.* showed that the Bax/Bcl-2 ratio determines the susceptibility of cells to apoptosis [92]. Thus, a low Bax/Bcl-2 ratio is associated with enhanced survival of BC cells and resistance to apoptosis, and vice versa. It has been proposed that diets rich in *n*-3 PUFA, such as fish and canola oil, reduces the abundance of Bcl-2 and up-regulates Bax expression to induce apoptosis, thereby reducing BC risk [27,93].

#### 3.3.4. PI3K/Akt, NF-κB

Besides Bcl-2, the PI3K/Akt pathway also plays an important role in cell apoptosis. Phosphatidylinositol 3-kinase (PI3K) is a heterodimeric lipid kinase that is composed of a regulatory and catalytic subunit that are encoded by different genes [93]. The primary consequence of PI3K activation is the generation of the second messenger PtdIns (3,4,5) P3 (PIP3) in the membrane, which in turn recruits and activates Akt, a downstream serine/threonine kinase [94]. Akt activation is a dual regulatory mechanism that requires translocation to the plasma membrane and phosphorylation at Thr308 and Ser473 [52,94,95]. Due to this mechanism, Akt functions as an anti-apoptotic signaling molecule. Thus, upregulation of phosphorylated Akt is relevant to tumor cell growth and resistance to cell apoptosis [6,87,96]. HER-2 overexpression constitutively activates survival and proliferation pathways by increasing activation of Akt, however, *n*-3 PUFA was found to either modulate total Akt expression or interact with Akt to down-regulate its phosphorylation [6,97]. Since Akt requires translocation to the plasma membrane for activation, it is possible that tumor cell membrane enrichment of *n*-3 PUFA might affect the phosphorylation of Akt that are recruited to the membrane for activation [96]. In addition, recent studies have demonstrated that PI3K/Akt promoted cell survival is mediated, in part, through the activation of the nuclear factor kappa-B (NF-κB) transcription factor [95,98,99]. NF-κB is a key regulator of genes involved in cell proliferation, migration, and angiogenesis [100,101]. In tumor cells, impaired regulation of NF-κB activation will lead to deregulated expression of the anti-apoptotic genes under the control of NF-κB [100]. For instance, NF-κB has been shown to inhibit the activity of p53, a tumor suppressor known to trigger apoptosis in cells with damaged DNA [102]. As a result, constitutive NF-κB expression may contribute to the development and progression of BC.

#### 3.3.5. Cell Proliferation Marker: Ki-67 and PCNA

Cell proliferation is another fundamental process integral to carcinogenesis. Ki-67 is a nuclear protein, and being widely used as a prognostic or predictive marker in BC and other malignant disease [103]. The human Ki-67 protein is present during all active phases of the cell cycle G(1), S, G(2), and mitosis, but is absent from resting cells G(0), which makes Ki-67 an excellent marker for determining the growth fraction of a given cell population [104]. Ki-67 immunohistochemical staining has been used as an index of tumor growth in numerous of cancer studies, especially prostate and breast carcinomas [26,87]. Treatment with ALA-rich flaxseed oil markedly lowered tumor burden in rats accompanied by reduced Ki-67 level [78,105].

Similarly to Ki-67, Proliferating Cell Nuclear Antigen (PCNA) is also considered a potential prognostic marker in BC [106]. PCNA is a ring-like nuclear protein which functions as the sliding clamp of DNA polymerases [107,108]. Thus, it is involved in DNA replication and repair machinery of the cell [109]. Expression of PCNA is a valid cell proliferation marker since the distribution of PCNA was found to occur during G1, S and G2 phase, but reaches low immunohistochemically detectable levels in M-phase of the cell cycle [106,108,109]. It has been shown that supplementation with *n*-3 PUFA reduces the percentage of proliferating tumor cells by decreasing the expression of PCNA [110].

The overall effect of high *n*-3 PUFA intake on cellular signaling process either inhibits cell proliferation or promotes cell apoptosis (Figure 2). The changes in gene expression, transcription factor activity and signaling transduction will be highlighted in the following sections.

## 4. The Effect of *n*-3 PUFA Mixtures on BC Development

### 4.1. Animal Studies

The inhibitory effects of *n*-3 PUFA on tumor growth have been well documented in rodent models of BC. Xenograft, transgenic and chemically induced methods are the three main approaches employed in rodent models.

#### 4.1.1. Breast Cancer Studies in Xenograft Rodent Models

Xenograft rodent models involve the transplantation of human BC cells into immunocompromised mice [78,111]. The type and concentration of dietary PUFA have profound influences on the growth rate of transplantable human BC in rodents (Table 3). In one study, MDA-MB-435 BC cells xenografted into athymic nude mice in order to compare intake of LA alone with diets containing LA and various proportions of EPA/DHA [20]. The diet rich in *n*-6 PUFA stimulated the growth and migration of human BC cells in mice, whereas diets supplemented with EPA or DHA exerted suppressive effects [20]. Similarly, Karmail *et al.* also found an inhibition of R3230AC mammary tumor growth in rat fed with Maxepa, a menhaden oil supplement that contains approximately 18% EPA and 12% DHA [112]. These observations were largely attributed to the high incorporation of *n*-3 PUFA into the tumor phospholipids, which further reduced pro-inflammatory eicosanoid synthesis from AA [20]. More recently, a diet supplemented with 3% w/w fish oil concentrate was shown to stimulate lipid peroxidation in the tumor cells, and thereby slowed down the tumor growth rate in athymic nude mice implanted with MDA-MB-231 [30]. Similar effects were observed in MCF-7 human breast cancer xenografts, although a higher percentage of fish oil (19% w/w) was required to decrease tumor volume in the nude mice [113]. These findings supported an earlier study showed that *n*-6/*n*-3 PUFA consumed in a low ratio resulted in prolonged tumor latency and reduced tumor growth rate in BALB/cAnN mice [114]. Therefore, diet enriched with *n*-3 PUFA can alter murine mammary tumorigenesis.

#### 4.1.2. Breast Cancer Studies in Transgenic Rodent Models

In transgenic mouse models, tumor growth can be initiated in two different ways: gain of function involving oncogenes responsible for cell proliferation or loss of function involving cell apoptotic pathways [115,116]. For example, the mouse mammary tumor virus (MMTV) promoter allows the cancer-causing virus to be activated and expressed in mammary tissue, leading to the development of mammary tumors [117]. Several studies have employed MMTV-neu related models and provided strong evidence for protective effects of *n*-3 PUFA towards HER-2 positive BC (Table 4).

One study examined mammary tumor development in MMTV-Her-2/neu mice fed menhaden fish oil from 7 weeks of age onwards. The tumor incidence was dramatically reduced as well as prolonged tumor latency [115]. Further, Yee *et al.* demonstrated that dietary *n*-3 PUFA downregulated COX-2 and Ki-67 to inhibit cell proliferation, further reducing atypical hyperplasia to prevent HER-2/neu mammary carcinogenesis at early stages [77]. Moreover, we showed that lifelong *n*-3 PUFA exposure can mitigate tumor development in mice expressing MMTV-neu(ndl)-YD5, a more aggressive HER-2- positive BC model [116]. In this study, MMTV-neu(ndl)-YD5 mice were crossed with fat-1 mice, yielding mice that were capable of endogenous synthesis of *n*-3 from *n*-6 PUFA and had the susceptibility to mammary tumor growth [116]. Thus, this study provided a direct evidence for a protective effect of *n*-3 PUFA via both complementary genetic and conventional dietary approaches [116]. In order to understand the dose-dependent effect of *n*-3 PUFA on mammary gland tumor development, Leslie *et al.* further demonstrated a dose-response relationship of 0%, 3% and 9% (w/w) menhaden oil on tumor burden reduction in MMTV-neu-YD5 mice [21]. In line with the previous study, the observed dose-dependent effects of *n*-3 PUFA were associated with a dose-dependent change in the fatty acid profile, reflecting a decreased *n*-6/*n*-3 ratio in the mammary glands and an increased of EPA and DHA in tumor phospholipid classes [21]. This is consistent with an earlier human clinical trial which showed dietary DHA and EPA supplementation caused a dose-dependent increase of *n*-3 PUFA in serum and breast adipose tissues of BC patients [118].

**Table 3:** *n-*3 PUFA and breast cancer risk: Xenograft rodent models.

Animal Model	*n-*3 PUFA Source	Feeding Period	Main Findings	Mechanism	Reference
Athymic nu/nu mice MDA-MB 231	3% w/w fish oil concentrate (10.2 g/kg EPA, 7.2 g/kg DHA, 3.0 g/kg ALA)	7-week (fed after tumor established)	↓ tumor growth rate ↑ effectiveness of doxorubucin	↑ EPA incorporation into tumor ↑ lipid peroxidation in tumor	[30]
Athymic nu/nu mice (NCr-nu/nu) MDA-MB 435	40 or 80 g/kg EPA, DHA	13-week (fed before transplantation)	↓ tumor growth, size ↓ tumor weight	↑ EPA, DHA in tumor phospholipids ↓ LA, AA in tumor phospholipids ↓ AA-derived eicosanoids	[20]
Inbred F44 rats R3230AC	5% marine oil supplementation (18% EPA, 12% DHA)	4-week (fed before transplantation)	↓ tumor weight, volume	↑ EPA, DHA, AA incorporation into tumor ↓ Prostaglandins 2 series	[112]
BALB/cAnN mice Mouse BC cell	10% or 20% w/w menhaden fish oil	7-week (fed before transplantation)	↑ tumor latency ↓ tumor growth rate	NA	[114]
Athymic nude mice MCF-7	19% w/w menhaden oil (1.9 g/kg ALA, 19.4 g/kg EPA, 24.3 g/kg DHA)	6 or 8-week (fed after tumor established)	↓ tumor volume	↑ lipid peroxidation in tumor	[113]

↑: increase; ↓: decrease; NA: not available.

**Table 4:** *n-*3 PUFA and breast cancer risk: transgenic rodent models.

Animal Model	*n-*3 PUFA Source	Feeding Period	Main Findings	Mechanism	Reference
MMTV-HER-2/neu	22.50 kcal% menhaden oil (15 g/kg EPA, 10.8 g/kg DHA)	28-week (fed before tumor development)	↓ atypical ductal hyperplasia ↓ cell proliferation prevented HER-2/neu at early stages	↓ Ki-67 expression ↓ COX-2 expression	[77]
MMTV-HER-2/neu	22.50 kcal% menhaden oil (15 g/kg EPA, 10.8 g/kg DHA)	52-week (fed before tumor development)	↓ tumor incidence and multiplicity ↑ tumor latency ↓ mammary gland dysplasia	NA	[115]
MMTV-neu (ndl)-YD5 × fat1	3% w/w menhaden oil (0.5 g/kg ALA, 4.1 g/kg EPA, 3 g/kg DHA)	20-week (lifelong treatment, fed before tumor development)	↓ tumor volume and multiplicity	↑ EPA, DHA and overall *n*-3 in mammary tissues ↓ *n*-6/*n*-3 ratio in tumor phospholipids	[116]
MMTV-neu (ndl)-YD5	3% w/w menhaden oil (0.5 g/kg ALA, 4.1 g/kg EPA, 3 g/kg DHA) 9% w/w menhaden oil (1.3 g/kg ALA, 12.4 g/kg EPA, 9 g/kg DHA)	20-week (lifelong treatment, fed before tumor development)	↓ tumor volume and multiplicity ↑ tumor latency (all in a dose-dependent manner)	↑ EPA, DPA in mammary tissues ↑ EPA, DHA in tumor phospholipids ↓ LA, AA, *n*-6/*n*-3 PUFA ratio in both mammary and tumor tissues in a dose-dependent manner	[21]

↑: increase; ↓: decrease; NA: not available.

Supplementing mice with 24% (w/w) menhaden oil delayed mammary tumor development by 15 weeks relative to mice fed the same amount of corn oil [119]. This result heightened the potential value of *n*-3 as an effective agent against BC, while the timing of exposure to *n*-3 PUFA will also influence future cancer risk [34]. Mammary gland research has identified critical periods of development, including early windows such as in utero, lactation and pubescence. The mammary gland undergoes rapid growth during these periods, and exposure to environmental agents may influence the long-term health of mammary tissue. Thus, supplement with *n*-3 PUFA in early life stages may protect against later BC development. In support of this, Su *et al.* conducted a study exposing rats to maternal high *n*-6 PUFA diet with or without fish oil supplementation during the perinatal period via maternal intake or during puberty or adulthood [29]. They found that fish oil intake during the perinatal period had a greater effect in preventing mammary tumors than fish oil supplementation in later life [29]. The decreased maternal serum estradiol levels in pregnant rats with fish oil supplementation was thought to play a role in reducing susceptibility to later BC development in the female offspring [29].

#### 4.1.3. Breast Cancer Studies in Chemically-Induced Rodent Models

Evidence from chemical-induced BC studies consistently supports the anti-cancer effect of *n*-3 PUFA. Induced mammary carcinoma in rats by the injection of carcinogenic chemicals such as 7, 12-dimethylbenz (α) anthracene (DMBA) and *N*-methyl-*N*-nitrosourea (MNU) have been widely used in various BC chemopreventive studies. When provided in the diet through menhaden or fish oil concentrates, the incorporated *n*-3 PUFA, EPA and DHA in particular, lower tumor incidence as well as retard tumor growth and metastasis of chemically-induced and transplantable mammary tumors (Table 5). Olivo *et al.* compared the effects of low- or high-, *n*-3 or *n*-6 PUFA exposure on mammary tumorigenesis in rats [110]. Feeding rats a low-fat *n*-3 diet significantly lowered the incidence of DMBA-induced mammary tumors compared to *n*-6 fed rats. This was accompanied by reduced cell proliferation and elevated lipid peroxidation. However, the high-fat *n*-3 diet resulted in a significant increase risk of mammary tumorigenesis relative to rats fed with *n*-6 diets [110]. Further, an *in vivo* study demonstrated that low-fat *n*-3 PUFA diets inhibited cell proliferation via activation of PPARγ and/or down-regulation of COX-2 and PCNA expression; whereas high fat *n*-3 PUFA diets stimulated cell proliferation and were positively associated with levels of phosphorylated Akt [110]. Similar suppressive effects of *n*-3 PUFA were observed in another DMBA-induced rat model [27]. Supplementation of EPA and DHA (from Maxepa) effectively suppressed cell proliferation, which was accompanied by down-regulation of Ki-67 and HER-2/neu positive expressions. Meanwhile, EPA and DHA induced cell apoptosis through modulating the expression of Bcl-2 and Bax in mammary tissue of Sprague-Sawley rats [26,27,28]. These findings suggest that gene-nutrient interactions are of a critical importance in the development of BC.

**Table 5:** *n-*3 PUFA and breast cancer risk: Chemically-induced rodent models.

Carcinogen	*n-*3 PUFA Source	Feeding Period	Main Findings	Mechanism	Reference
MNU	Fish oil 2%–10% w/w *n*-3 PUFA in diet	18-week (at the same time as MNU administration)	Absolute *n*-3 diet: ↓ body weight, no tumor occurrence (10% w/w *n*-3 PUFA) 1:1*n*-6/*n*-3 diet ↓ tumor incidence and multiplicity (5% w/w *n*-3 PUFA)	↑ EPA, DHA in mammary ↓ FAS, COX-2, 5-LOX	[64]
MNU	Fish oil concentrate Low *n*-6/*n*-3 = 1:14.6 High *n*-6/*n*-3 = 1:0.7	2-week (at the same time as MNU administration)	Low *vs.* high ratio *n*-6/*n*-3 PUFA diet: ↓ tumor incidence (21%), ↓ tumor multiplicity (30%), tumor burden (80%) ↑ apoptotic index (129%)	↓ Ki-67 ↑ Bax, Bax/Bcl2, PPARγ ↓ NF-κB p65, pAkt, IGF-IR	[87]
MNU	EPA/DHA alone: 95 g/kg EPA/DHA EPA + DHA: 47.5 g/kg EPA + 47.5 g/kg DHA	20-week (at the same time as MNU administration)	DHA alone *vs* EPA + DHA *vs* EPA alone: ↓ tumor incidence: 23%, 73%, 65% ↓ tumor multiplicity: 0.23, 1.67, 1.59 DHA is more effectively than EPA	NA	[120]
DMBA	Maxepa (fish oil concentrate): 90 mg EPA + 60 mg DHA per day	24-week study 35-week study (before DMBA injection)	↓ DNA single-strand breaks ↓ cell proliferation	↓ Ki-67, Her-2/neu	[26]
DMBA	Maxepa: 90 mg EPA + 60 mg DHA per day	24-week study 35-week study (before DMBA injection)	↓ tumor incidence (23%), tumor multiplicity (42%) ↑ cell apoptosis ↓cell proliferation	↓ Bcl-2 ↑Bax ↑ p53	[27,28]
DMBA	Fish oil (0.5%ALA, 16% EPA, 1.2% DPA, 8% DHA in fish oil)	NA	↓ tumor incidence with fish oil consumption: adulthood < in utero < puberty < perinatal ↓ tumor multiplicity with fish oil consumption: adulthood > puberty > perinatal > in utero	↓ maternal serum estradiol	[29]
DMBA	Menhaden oil Low-fat *n*-3 PUFA diet: 4.6 g/kg EPA + 3.2 g/kg DHA High fat *n*-3 PUFA diet: 9.1 g/kg EPA + 6.3 g/kg DHA	20-day (before DMBA injection)	Low *n*-3 diet: ↓ tumor incidence ↓ TEBs ↓ cell proliferation ↑ cell apoptosis; High *n*-3 diets exert opposite effects	Low *n*-3 diet: ↓ COX-2, PCNA ↑ PPARγ ↑ lipid peroxidation High *n*-3 diet: ↑ pAkt ↑ lipid peroxidation	[110]

↑: increase; ↓: decrease; NA: not available.

It has been shown that the different dietary fatty acid composition and *n*-6/*n*-3 PUFA ratios can diversely influence the occurrence and progression of BC [8,32,64]. Wei *et al.* fed MNU-induced rats with various *n*-6/*n*-3 PUFA ratios and demonstrated the 1:1 ratio of *n*-6/*n*-3 PUFA in diet was more effective in the prevention of mammary tumor development when compared with diets higher in *n*-6 PUFA [64]. Replacement of *n*-6 by *n*-3 PUFA in diets, reflected an increase in EPA and DHA in mammary tumor tissue, which subsequently downregulated the expression of lipid metabolic-related genes and inhibited cell proliferation [64]. In agreement with this study, Jiang *et al.* reported that low *n*-6/*n*-3 PUFA ratio (1:14.6) caused 80% reduction in tumor burden and 30% decrease in tumor multiplicity in the same rodent model compared to a high *n*-6/*n*-3 PUFA ratio (1:0.7). These observations were mainly mediated by downregulating NF-κB, pAkt and IGF-IR, and increased activity of PPARγ [87].

### 4.2. Cell Culture Studies

It has been well established that *n*-3 PUFA can suppress the development of cancers by inhibiting cellular proliferation and inducing apoptosis. Cell culture studies investigating the effects of *n*-3 PUFA on murine and human BC cells provide important insights into the mechanisms underlying this inhibitory effect. Although there are few studies describing the individual effects of ALA, EPA and DHA *in vitro*, the available data consistently show that *n*-3 PUFA have direct growth inhibitory effects on several BC cells lines (Table 6).

MDA-MB-231 human BC cell line was used to investigate the effect of various classes of fatty acids (*n*-3, *n*-6 and *n*-9 PUFA) [121]. EPA and DHA exhibited a dose-dependent inhibition of cell growth, whereas LA and oleic acid (OA) stimulated cell growth at very low concentration [121]. Some *in vitro* studies have not included the essential *n*-6 PUFA (*i.e.*, LA) in the growth medium, which would be present *in vivo* and is required for mammary tumorgenesis in animals. These studies are not able to explain the tumor growth inhibition by *n*-3 PUFA in the presence of abundant LA. Schley *et al.* examined the inhibitory effects of *n*-3 PUFA on BC *in vitro* in the presence or absence of LA [22,96]. It was demonstrated that EPA and DHA induced apoptosis and increased DNA fragmentation in MDA-MB-231 cells when provided in combination with LA. Similar effects were observed in the MCF-7 BC cell line [71,86,105,122]. Barascu *et al.* revealed that EPA and DHA decreased MCF-7 cell growth and increased the fraction of apoptotic cells in a concentration-dependent manner, and particularly, a higher efficiency noted for DHA [86]. Specifically, they demonstrated that *n*-3 PUFA inhibited cell proliferation by lengthening the cell cycle between the G2/M transition [86]. In accordance with this finding, a previous study demonstrated that DHA induced marked G2-M and G1-S arrest of the MCF-7 cells [123]. Furthermore, treatment with EPA and DHA was also shown to induce cell differentiation and increase lipid peroxidation product levels in MCF-7 cells [105,124].

**Table 6:** *n-*3 PUFA and breast cancer risk: cell culture studies.

Cell Type	*n-*3 PUFA Source	Main Finding	Mechanism	Reference
MDA-MB-231	EPA/DHA alone: 75 μM or 100 μM EPA + DHA combination: 45 μM EPA + 30 μM DHA or 60 μM EPA + 40 μM DHA (in presence/absence of LA)	↓ cell viability, cell proliferation ↑ DNA fragmentation, cell apoptosis DHA was more potent than EPA	↓ pAkt ↓ NF-κB and DNA binding activity	[96]
MDA-MB-231	0.5–2.5 μg/mL of EPA, DHA (1.7–8.2 μM EPA, 1.5–7.6 μMDHA)	↓ tumor cells growth (DHA > EPA, dose-dependent)	↓ LA composition in cell lipids ↓ AA-derived eicosanoid synthesis	[121]
MDA-MB-231	EPA/DHA alone: 75 μM or 100 μM EPA + DHA combination: 45 μM EPA + 30 μM DHA or 60 μM EPA + 40 μM DHA (in presence/absence of LA)	↓cell growth (48%–62%)	↑ EPA, DHA, DPA and total *n*-3 in lipid rafts ↓ EGFR levels ↑ pEGFR	[22]
MDA-MB-231 MCF-7	EPA (230 μM), DHA (200 μM)	↓ cell viability ↑ cell apoptosis	↓ Bcl-2 ↑pro-caspase-8 ↓ pEGFR ↓ EGFR (only DHA) ↓ AA ↑ EPA, DPA, DHA in total cell lipids	[71]
MDA-MB-231 MCF-7	3–100 μM of EPA, DHA	At 50 μM EPA, 30 μM DHA ↑ cell apoptosis ↓ cell growth At 50 μM EPA, DHA ↑ G2/M duration DHA was more potent than EPA	↓ phosphorylation of cyclin B1 ↓ activity of CDK1-cyclin B1	[86]
MCF-7	100 μM of EPA, DHA	↓ cell growth (30% by EPA, 54% by DHA) ↑ cell differentiation (30% by EPA, 65% by DHA) No significant effects on cell apoptosis and cell cycle DHA was more potent than EPA	↑ PPARγ (DHA only)	[125]
MCF-7 MCF-10A	6–30 μM of ALA, EPA, DHA	All *n*-3 PUFA ↓ MCF-7 cell growth (EPA, DHA > ALA, dose-dependent) AA ↓ MCF-7 cell growth (similar as ALA)	NA	[122]
ER+ and ER− cells	20 μg/mL of ALA, EPA, DHA (72 μM ALA, 66 μM EPA, 61 μM DHA)	EPA, DHA ↓ cell proliferation (all cell lines) ALA ↓ estrogen independent BC cell proliferation	↑ lipid peroxidation	[124]

↑: increase; ↓: decrease; NA: not available

Chajes *et al.* examined the effect of ALA, EPA, and DHA on the proliferation of human estrogen-positive (ER+) and estrogen-negative (ER−) BC cell lines [124]. EPA and DHA displayed a significant inhibitory effect on the proliferation of all types of tumor cells, whereas ALA significantly inhibited cell growth in (ER−) MDA-MB-231 and HBL-100 human breast tumor cells but not in (ER+) MCF-7 cells [124]. The efficiency of this inhibitory effect was found to be correlated with the generation of lipid peroxidation products [124]. Consistently, another *in vitro* study identified a dose-dependent inhibitory effect of ALA, EPA, and DHA on MCF-7 cells; however, the cells were dramatically inhibited by EPA and DHA, and moderately inhibited by ALA [122].

Rapid tumor cell proliferation is a critical feature for tumor aggressiveness. Treatment with LA and OA increased MDA-MB-231 cell proliferation through production of pro-inflammatory prostaglandins and leukotrienes [121]. EPA and DHA could mimic the effect of indomethacin, an inhibitor of both COX and LOX, which in turn attenuated BC cell proliferation by inhibiting *n*-6 PUFA related eicosanoid synthesis [126]. In addition, treatment with EPA and DHA inhibited EGFR phosphorylation and diminished EFGR levels in lipid rafts [22,71]. These observations were due to the incorporation of *n*-3 PUFA into BC cell membrane, which subsequently altered membrane structure, signal transduction and function of BC cells [71]. Moreover, *n*-3 PUFA can exert anti-proliferative effects by limiting cell cycle progression, attributed to an inhibition of CDK1-cyclin B1 complex, a master regulator required for the initiation of mitosis [86]. Furthermore, increasing evidence demonstrated that *n*-3 PUFA can inhibit cell proliferation and increase cell differentiation by activating PPARγ, although this effect was only observed with DHA treatment [105].

Dysregulation of apoptosis is also a hallmark of cancer cells, and thus agents that activate apoptosis are highly desired. Treatment with EPA and DHA inhibited phosphorylation of Akt as well as the NF-κB DNA binding activity [96]. This represents a novel mechanism by which *n*-3 PUFA induce apoptosis in MDA-MB-231 cells [96]. Additionally, it has been shown that *n*-3 PUFA decreased expression of Bcl-2 and increased activity of pro-caspase-8, an apoptosis effector enzyme [71]. As a result, *n*-3 PUFA can inhibit BC development *in vitro* by both suppressing tumor cell proliferation and inducing tumor cell death.

## 5. The Effect of Individual *n*-3 PUFA on BC Development

### 5.1. ALA and BC

#### 5.1.1. Inefficient Conversion from ALA to EPA and DHA

α-Linolenic acid (18:3*n*-3; ALA) is the major *n*-3 PUFA in the Western diet. Typical consumption of ALA in Europe, Australia and North America ranges between 0.6 and 1.7 g per day in men and 0.5–1.4 g per day in women [58]. This is about 10-fold lower than the consumption of *n*-6 PUFA. ALA, regarded as the precursor for long-chain PUFA, can be converted to EPA (20:5*n*-3), DPA (22:5*n*-3) and DHA (22:6*n*-3) by the pathway shown in Figure 1. Whether the essentiality of ALA in the diet primarily reflects the activity of ALA itself or of long-chain PUFA synthesized from ALA is a matter of debate. The concentration of ALA in plasma phospholipids, cells and tissues is found to less than 0.5% of total fatty acids [127]. Although the dietary intake of EPA and DHA are approximately 10-fold lower than those of ALA in North America, the concentrations of these long-chain PUFA in plasma, cell and tissue phospholipids are greater than those of ALA [127]. This apparent mismatch between dietary intakes and levels of incorporation further suggests that the primary biological role of ALA is for EPA and DHA synthesis. However, it is also possible that the low concentration of ALA may be due to negative selection in the incorporation of ALA into blood and cell membrane lipid pools [58]. Since consumption of EPA and DHA show a strong inverse association with the risk of BC, this raises the question of whether conversion of ALA to EPA and DHA in humans is a viable alternative to dietary sources of these long-chain PUFA. The majority of human studies estimate that ALA supplementation in human adults generally lead to an increase in EPA and DPA, but have little or no effect on DHA content [128,129,130,131,132]. Conservatively, Pawlosky *et al.* estimated the overall efficiency of conversion from ALA was 0.2% to EPA, 0.13% to DPA and 0.05% to DHA [129]. This inefficient conversion is due to the first rate limiting reaction catalyzed by Δ6-desaturase [58]. As a result, the extent of ALA conversion to EPA and DHA is inefficient and limited. Thus, ALA may not be considered as an effective alternative source to fish for providing EPA and DHA.

#### 5.1.2. Individual Effect of ALA on Breast Cancer

Data derived from epidemiological and observational studies suggest that ALA present in the Western diet has protective effects in BC (Table 7 and Table 8). Two case control studies compared the fatty acid composition in the adipose breast tissue from women with invasive non-metastatic breast carcinoma and women with benign breast disease [45,133]. Low ALA content in adipose tissue was found to be associated with an increased risk of BC. This observation was consistent with a previous cohort study on 121 BC patients, which demonstrated a link between a low level of ALA in adipose breast tissue and increased risk of metastatic development [134]. In particular, the ratio of *n*-6/*n*-3 PUFA was also shown to be positively correlated with BC in these patients, highlighting the role of *n*-3 and *n*-6 PUFA balance in BC.

Dietary supplementation of ALA reduced the growth of established mammary tumors in chemically-induced rats and xenograft rodent models. In OVX athymic mice with high circulating estrogen level, flaxseed oil and its high ALA content, attenuated (ER+) MCF-7 breast tumor growth by reducing cell proliferation and increasing apoptotic index [78]. This effect was probably due to the downregulation of tyrosine kinase receptors such as EGFR and HER2, with a subsequent reduction in pAkt. Compared to corn oil, consumption of ALA-rich flaxseed oil tends to modify the *n*-6/*n*-3 PUFA ratio by increasing serum ALA, EPA and DHA concentrations. This provides evidence that ALA absorption and conversion may contribute to the observed tumor reducing effect in the flaxseed oil diet [78]. Further, an* in vitro* study showed that pure ALA inhibited MCF-7 cell proliferation by 33%, which was in accordance with the* in vivo* reduction in palpable tumor growth (33%) [78]. This suggests that ALA itself, rather than the generated EPA and DHA, exerts this anti-tumorigenic effect [78]. Another* in vivo* study was designed to elucidate which component(s) of flaxseed (lignan or ALA) was responsible for enhancing tamoxifens effect on reducing the growth of established MCF-7 breast tumors at low circulating estrogen levels [105]. ALA-rich flaxseed oil had a stronger effect in reducing the palpable tumor size of tamoxifen-treated tumors compared with lignan-treated mice [105]. More importantly, ALA was found to downregulate HER2 expression, and subsequently modulate growth factor-mediated signaling pathways by repressing IGF-1R and Bcl-2 [105].

**Table 7:** Individual role of ALA, EPA and DHA on BC.

*n-*3 PUFA	Amount of Fatty Acid	Effect	Mechanism	Reference
ALA	NA	Moderate decrease BC risk	NA	[45]
	~22.8 g of ALA per kg diet	Reduced tumor cell proliferation	Inhibited HER2, EGFR expression	[78]
	~22.8 g of ALA per kg diet	Inhibited MCF-7 cell proliferation		[78]
	~11 g ALA per kg diet	Reduced tumor incidence and burden	Increased BAX/Bcl-2 ratio	[93]
	10.6 g ALA per kg diet	Decreased tumor growth rate	Inhibited HER2 expression	[105]
	72 μM ALA	Moderate inhibited ER-negative cell proliferation, not affect MCF-7	NA	[124]
	30 μM of ALA	Slightly inhibited MCF-7	NA	[122]
	NA	Inversely associated with BC risk	NA	[133]
	NA	Inversely correlated with metastasis development	NA	[134]
	55.9 g ALA per kg diet	Reduced tumor growth and metastasis	NA	[135]
	8 g ALA per kg diet	Decreased tumor growth rate	NA	[136]
	10 g ALA per kg diet	Reduced tumor burden and increased survival rate	NA	[137]
	2.5-40 μM of ALA	enhanced cytotoxic effects of Trastuzumab (at 10 μM of ALA)	Down-regulated HER2 (at 20 μM of ALA)	[138]
	10 μM of ALA	Diminished proteolytic cleavage of the extracellular domain of HER2	Inhibited HER-2 activity	[139]
	~21.2 g of ALA per kg diet	Minimal inhibited tumor growth w/wo Trastuzumab	NA	[140]
	52.8 g of ALA per kg diet	Inhibited mammary tumor development	NA	[141]
EPA	40–80 g of EPA per kg diet	Slowed down tumor growth, reduced tumor burden	Decreased AA derived-eicosanoid	[20]
	3–100 μM of EPA	Induced BC cell apoptosis (at 50 μM of EPA)	NA	[86]
	40–200 μM of EPA	Restored the growth inhibitory effect of Tamoxifen (at 40 μM of EPA)	Decreased pAkt (at 20 μM of EPA)	[97]
	20–80 g of EPA per kg diet	Inhibited the development of lung metastasis	NA	[126]
	100 μM of EPA	Inhibited MCF-7 cell growth	NA	[125]
	40 μM of EPA	Induced apoptosis, inhibited cell proliferation, arrested cell cycle at G0/G1	down-regulated Bcl-2 expression	[142]
	95 g of EPA per kg diet	Reduced KPL-1 cell proliferation rate and metastasis	NA	[143]
	42 g of EPA per kg diet	Suppressed cell proliferation in MCF-7 xenografts in rats	NA	[144]
	50 μM of EPA	Increased PPARγ at mRNA level	NA	[145]
	0–200 μM of EPA	Inhibited MCF-7 cell growth (at 60 μM of EPA)	NA	[146]
DHA	120 μM of DHA	Decreased cancer cell viability, enhanced the cytotoxic activity of taxanes	Decreased the expression of Her-2/neu	[5]
	100 μM of DHA	Disrupted lipid rafts, induced apoptosis in HER-2 overexpressing cells	Decreased Akt activity and FAN	[6]
	100 μM of DHA	Decreased MDA-MB-231 cell proliferation, enhanced EGFR inhibitors	Altered EGFR phosphorylation and localization	[56]
	0–200 μM of DHA	Reduced MCF-7 cell viability and DNA synthesis (at 25 μM of DHA)	Increased lipid peroxidation, capase 8 activation	[146]
	20 or 100 μM of DHA	Inhibited MDA-MB-231 cell proliferation, promoted nuclear condensation	Increased caspase-3 activity (at 100 μM of DHA)	[147]
	10–160 μM of DHA	Inhibited MCF-7 cell growth and induced apoptosis (at 40 μM of DHA)	Downregulated Bcl-2, increased Bax/Bcl-2 ratio	[148]
	270 μM of DHA	50% inhibitory KPL-1 cell growth after 72 h treatment	Downregulated Bcl-2, increased Bax/Bcl-2 ratio	[149]
	40 g of DHA per kg diet	Decreased tumor growth rate and final tumor weight, increased apoptosis	Reduced tumor PGE2, decreased Ki-67	[150]
	32 g of DHA per kg diet	Reduced tumor incidence	Increased BRCA1 at protein level	[151]
	30 μM of DHA	50% inhibitory MCF7 cell growth after 96 h treatment	Increased BRCA1/2 at transcriptional level	[152]
	NA	Increased response of the tumor to chemotherapies, increased survival rate		[153]

NA: not available.

**Table 8:** Individual effect of ALA, EPA and DHA on different types of BC.

BC Cell Type	ALA	EPA	DHA
MDA-MB-231 (ER−)	✓	✓	✓
MDA-MB 435 (ER−)	NA	✓	✓
MCF-10A (ER−)	—	✓	✓
HBL-100 (ER−)	✓	✓	✓
MCF-7 (ER+)	—	✓	✓
ZR-75 (ER+)	—	✓	✓
T-47-D (ER+)	—	✓	✓
SK-Br3 and BT-474 (HER-2/neu positive)	✓	NA	✓

✓ have significant inhibitory effect on cell proliferation; — slightly inhibit the cell growth; NA: not available.

Remarkably, long-term changes in dietary ALA and LA ratio significantly affect mammary tumor outcomes. Feeding BALB/c mice with ALA-rich linseed oil inhibited the development of mammary tumors compared to mice on corn oil diets [135]. In a recent study, use of canola oil (10% ALA) instead of corn oil (1% ALA) in the diet of MDA-MB-231 implanted mice reduced tumor growth rate [136]. Progression to apoptosis is associated with a balance between pro-apoptotic Bax and anti-apoptotic Bcl-2. Mice exposed to canola oil had an increased ratio of Bax/Bcl-2, which was responsible for the apoptosis of defective epithelial cells [93]. Furthermore, the maternal diets have a life-long influence on development of BC in the daughter. Substitution of corn oil with canola oil in the maternal diet increased *n*-3 PUFA incorporation into mammary glands, which in turn delayed occurrence of mammary tumors and increased tumor cell apoptosis in offspring [93]. Elsewhere, maternal replacement of dietary soybean oil with canola oil significantly lowered the burden of MNU-induced mammary tumors, along with increased survival rate in the offspring [137]. As a result, maternal ALA supplementation brings about a stable epigenetic imprint of genes that are involved in the development and differentiation of the mammary gland, which are passed on to female offspring where they exert a protective effect against BC.

Notably, the protective role of ALA in BC was inconclusive in* in vitro* studies. In BT-474 and SkBr-3 cancer cells that naturally amplify the HER-2 oncogene, exogenous supplementation with ALA significantly suppressed HER-2 mRNA expression, thereby reducing the probability of activation that leads to tumor growth [138]. Moreover, ALA co-exposure was reported to synergistically enhance trastuzumab (an anti-cancer therapy) efficacy in HER2-overexpression BC cells [139]. However, in a separate study, ALA exerted minimal tumor-reducing effects in the presence of trastuzumab [140]. In addition, some studies had difficulties in characterizing the role of ALA in estrogen dependent and independent BC cell lines [122,124]. This suggests a variable effect of ALA on cell proliferation depending on the cell line assessed.

Overall, these findings suggest that diets rich in ALA can inhibit mammary tumor development in animals and* in vitro*, however, it cannot be ruled out that some of the effects are due in part to conversion of ALA to EPA and DHA, albeit limited.

### 5.2. Individual Effect of EPA on Breast Cancer

Fish oil is a mixture of EPA and DHA, which have been previously shown to have protective effects against BC. However, whether EPA and DHA differentially or similarly affect BC has not yet been determined. In support of the independent effect of EPA on BC, several* in vitro* studies consistently demonstrated the ability of EPA to induce apoptosis in human BC cells (Table 7 and Table 8). Chiu* et al.* demonstrated that 40 μM EPA induced cell apoptosis through inhibition of anti-apoptotic regulator proteins, such as Bcl-2 [142]. Moreover, Akt was also shown to be susceptible to EPA [97]. Treatment with EPA lowered both total and phosphorylated Akt content in transfected MCF-7 cells that overexpressing constitutively active Akt [97]. Additionally, co-treatment with EPA enhanced the growth inhibitory response to tamoxifen in MCF-7 cells, thus suggesting that EPA may be useful as a nutritional adjuvant in the treatment of BC [97].

Diets rich in EPA have also been shown to inhibit the growth of spontaneous or transplanted mammary carcinomas in animal models and human clinical trials. EPA was observed to slow tumor growth and reduced metastasis in mice implanted with KPL-1 human BC cells [143]. In a separate study, when compared with LA diet, intake of EPA significantly inhibited tumor cell proliferation and development of lung metastasis in mice with induced mammary tumorigenesis [20,126]. These inhibitory effects were attributed to high incorporation of EPA into tumor phospholipids, and subsequently, disrupting inflammatory eicosanoid biosynthesis from AA [20]. More recently, EPA was found to regulate cell proliferation in MCF-7 xenografts via an inhibitory G protein-coupled receptor-mediated signal transduction pathway [144]. Furthermore, a human clinical study demonstrated a strong positive correlation between plasma EPA concentrations and PPARγ mRNA levels in adipose tissue of obese subjects [145]. Since activators of PPARγ are known to inhibit cell proliferation and tumorigensis, up-regulation of PPARγ gene expression by EPA might be a potential mechanism of action. These findings suggest that EPA can independently act to inhibit the development and progression of human BC.

### 5.3. Individual Effect of DHA on Breast Cancer

Several independent reports have shown that DHA can inhibit mammary carcinoma development and progression (Table 7 and Table 8). However, the specific mechanisms underlying these protective effects of DHA remain to be determined.

HER-2 signaling is central to many processes involved in cellular proliferation and survival [6]. Treatment with DHA alone* in vitro* was shown to be effective in disrupting lipid rafts in HER-2 overexpressing cells, inhibiting HER-2 activity and its downstream signaling molecules (Akt and FAS,* etc.*), and consequently led to cell death [6]. Menendez* et al.* demonstrated that exogenous supplementation with DHA was able to downregulate HER-2/neu oncogene expression in SK-Br3 and BT-474 human BC cells [5]. This supports the therapeutic potential of DHA supplementation in the treatment of HER-2 positive BC. On the other hand, recent evidence suggests that DHA itself is capable of decreasing EGFR localization in the lipid rafts of the MDA-MB-231 BC cell line [56]. Moreover, DHA supplementation was reported to significantly enhance the efficacy of EGFR inhibitors, which provides strong evidence for the potential development of combination therapies targeting EGFR [56]. The pre-exposure with DHA has been shown to synergistically enhance the cytotoxicity of antimitotic drugs including Taxane and Taxol against highly metastatic BC cells [5]. This was attributed to the incorporation of DHA into cellular lipids, and subsequently altered membrane fluidity and function, thereby increasing drug intake [5]. In agreement with this, a recent human clinical trial demonstrated that addition of DHA into chemotherapy increased survival in metastatic BC patients [154].

A number of earlier studies have suggested that the anti-cancer property of DHA is attributable to its ability to inhibit cell growth and induce apoptosis. Kang* et al.* demonstrated that DHA induced apoptosis in MCF-7 cells through a combination of pathways [146]. Mechanistically, it was largely due to increased lipid peroxidation, followed by accumulation of reactive oxygen species in cancer cells and higher oxidative stress, ultimately resulting in cell apoptosis [146]. It was also possible that the elevated intracellular levels of DHA stimulated activation of apoptosis effector enzyme, such as caspase-8 and caspase-3, and thus inducing apoptotic cell death [146,147]. Furthermore, Chiu* et al.* found that pure DHA inhibited growth of MCF-7 cells [148]. Although DHA did not affect pro-apoptotic Bax protein, it induced the downregulation of anti-apoptotic Bcl-2 gene expression time-dependently, and thus increasing the Bax/Bcl-2 ratio [148]. DHA has also shown to suppress KPL-1 cell growth* in vitro*, accompanied by downregulation of Bcl-2 [149]. Since the ratio of Bax/Bcl-2 is positively associated with apoptotic activity, the regulation of Bax and Bcl-2 can be considered an important step in the apoptotic actions of DHA.

Animal studies also support the anti-cancer role of DHA in BC. Dietary supplementation of pure DHA was showed to suppress tumor development in both chemically-induced carcinoma and xenograft rodent models [150,151]. Low level DHA administration markedly reduced tumor growth rates and tumor weight compared with rats fed LA [150]. These observed suppressive effects of DHA resulted from diminished tumor eicosanoid concentrations and decreased cell proliferation [150]. DHA has also been shown to decrease mammary tumor incidence coinciding with a 60% increase in BRCA1 protein, a major tumor suppressor [151]. In complement, DHA supplementation was shown to increase BRCA1 at the transcriptional level [152]. The correlation between the* in vitro* and* in vivo* observations adds weight to DHA’s potential mechanism and supports a beneficial role for DHA against BC.

Altogether, ALA, EPA and DHA have various effects against mammary tumor development* in vivo*. In terms of* in vitro* studies, EPA and DHA individually exert an inhibitory effect on the proliferation of almost all types of tumor cells* in vitro*; whereas ALA only has effects on ER− and HER-2 positive BC cells (Table 8). As little as 50 μM of EPA or 30 μM of DHA can dramatically reduce tumor cell viability, while 72 μM of ALA only cause moderate inhibition on ER− cell proliferation. However, it is not appropriate to compare the effective dosage of individual *n*-3 PUFA from different type of studies, since the amount of tumor cells and the duration of treatment are different. Additional experimental studies are needed to compare the efficacy of each individual *n*-3 PUFA under the same conditions.

## 6. Plant-Derived *n*-3 (ALA) *vs.* Marine-Based *n*-3 (EPA, DHA)

The potential health benefits of *n*-3 PUFA have been examined in various types of studies. However, the relative potency of plant-based *n*-3* versus* marine *n*-3 PUFA, as well as EPA* versus* DHA in inhibiting tumor growth remains unclear. There are only two studies that have compared the efficacy between EPA and DHA [120,155]. The first study compared the ability of dietary EPA or DHA to suppress MNU-induced mammary carcinogenesis in a rat model [120]. Although treatment with DHA or EPA alone was not as effective in combination, DHA was more potent in delaying tumor onset and reducing tumor multiplicity relative to EPA [120]. In accordance, several BC cell line studies indicated that DHA was more effective in inhibiting MDA-MB-231 and MCF-7 cell proliferation and invasion than EPA at the same concentration [86,96,121,125,155].

In terms of the efficacy of plant-based *n*-3 PUFA, there has not yet been a human clinical study directly comparing ALA* versus* EPA or DHA in BC. To the best of our knowledge, only two cell culture studies have examined the effect of different types of *n*-3 PUFA. ALA was less effective compared with EPA or DHA, but ALA had moderate inhibitory effects in some BC cell lines [122,124]. This may be due to the conversion of ALA to EPA and DHA which would lower the amount of ALA incubated with cancer cells. In addition, the lower incorporation of ALA into the cellular lipid pool may also be a confounding factor.

## 7. Conclusions

In summary, the present review has assessed the anticancer effects of *n*-3 PUFA when consumed or treated individually, as well as in *n*-3 PUFA mixtures. ALA, EPA and DHA can differentially inhibit mammary tumor development by changing the cell membrane fatty acid composition, suppressing AA-derived eicosanoid biosynthesis and influencing signaling transcriptional pathways to inhibit cell proliferation and induce apoptosis. This review also provided evidence for using *n*-3 PUFA as a nutritional intervention in the treatment of BC to enhance conventional therapeutics, or potentially lowering effective doses.

Overall, in order to provide definitive recommendations, additional human studies are required. Long term studies tracking fish or *n*-3 PUFA intake are needed to demonstrate a role for *n*-3 PUFA in prevention. Also, additional clinical trials are needed to evaluate the effect *n*-3 PUFA on BC outcomes. Nevertheless, evidence does not indicate harm and all forms of *n*-3 PUFA may be included in a healthy diet.
